# Morphological and physiological changes in *Lentilactobacillus hilgardii* cells after cold plasma treatment

**DOI:** 10.1038/s41598-020-76053-x

**Published:** 2020-11-03

**Authors:** Iwona Niedźwiedź, Wojciech Juzwa, Krzysztof Skrzypiec, Tomasz Skrzypek, Adam Waśko, Michał Kwiatkowski, Joanna Pawłat, Magdalena Polak-Berecka

**Affiliations:** 1 Department of Microbiology, Biotechnology and Human Nutrition, University of Life Sciences in Lublin, Skromna 8, 20-704 Lublin, Poland; 2 Department of Biotechnology and Food Microbiology, Faculty of Food Science, Poznan University of Life Sciences, Wojska Polskiego 48, 60-627 Poznan, Poland; 3 Analytical Laboratory, Maria Curie-Skłodowska University, M. Curie-Skłodowska Square 3/22, 20-031 Lublin, Poland; 4 Laboratory of Confocal and Electron Microscopy, Interdisciplinary Research Center, John Paul II Catholic University of Lublin, Lublin, Poland; 5 Faculty of Electrical Engineering and Computer Science, Lublin University of Technology, Nadbystrzycka 38A, 20-618 Lublin, Poland

**Keywords:** Biological techniques, Microbiology

## Abstract

Atmospheric cold plasma (ACP) inactivation of *Lentilactobacillus hilgardii* was investigated. Bacteria were exposed to ACP dielectric barrier discharge with helium and oxygen as working gases for 5, 10, and 15 min. The innovative approach in our work for evaluation of bacterial survival was the use in addition to the classical plate culture method also flow cytometry which allowed the cells to be sorted and revealed different physiological states after the plasma treatment. Results showed total inhibition of bacterial growth after 10-min of ACP exposure. However, the analysis of flow cytometry demonstrated the presence of 14.4% of active cells 77.5% of cells in the mid-active state and 8.1% of dead cells after 10 min. In addition, some of the cells in the mid-active state showed the ability to grow again on culture medium, thus confirming the hypothesis of induction of VBNC state in *L .hilgardii* cells by cold plasma. In turn, atomic force microscopy (AFM) which was used to study morphological changes in *L. hilgardii* after plasma treatment at particular physiological states (active, mid-active, dead), showed that the surface roughness of the mid-active cell (2.70 ± 0.75 nm) was similar to that of the control sample (2.04 ± 0.55 nm). The lack of considerable changes on the cell surface additionally explains the effective cell resuscitation. To the best of our knowledge, AFM was used for the first time in this work to analyze cells which have been sorted into subpopulations after cold plasma treatment and this is the first work indicating the induction of VBNC state in *L. hilgardii* cells after exposure to cold plasma.

## Introduction

Wine is one of the most frequently consumed alcoholic beverages in the world^1^. Due to the presence of alcohol as well as the low pH and compounds with antibacterial activity, wine is considered an unfavourable environment for the development of microflora. However, some microorganisms have developed a number of adaptive mechanisms that allow them to survive the unfavourable conditions prevailing in wine at particular stages of production, thus adversely affecting its physicochemical properties. This group includes mainly *Brettanomyces* yeasts, lactic acid bacteria (LAB), and acetic acid bacteria^2,3^. LAB are the most numerous group of bacteria in grape must and wine. In wine production, one of the main risks arising from the metabolism of lactic bacteria is the synthesis of ethyl carbamate. This compound has carcinogenic properties. Moreover, LABs are the main producers of biogenic amines (BA) such as histamine, tyramine, phenylethylamine, and spermidine. Biogenic amines are responsible for lowering the sensory quality of wine, but also their presence can be dangerous to consumer health. An example is histamine, which can cause ailments such as vomiting, headaches, and rash^4,5^. Extensive research conducted by Rodas et al. (2005) showed that the majority of isolates of LAB in wine belongs to *Lentilactobacillus hilgardii* species^6,7^. The strains of this species were found in wine with 20% ethanol content^6–8^. The aforementioned high resistance of *L. hilgardii* strains to high alcohol concentrations and other conditions in wine is a huge problem in winemaking because elimination thereof from the product is difficult. This is related to the metabolic characteristics and structure of the bacterial cell membrane. The presence of S-layer proteins on the cell surface and long unsaturated fatty acids (C19) in the direct membrane increases the resistance of the bacteria to alcohol. In the context of protection against adverse conditions, the S-surface layer plays an important role due to its stability at various pH and resistance to temperature, radiation, and proteolysis^9^. In addition, as shown by recent studies, *L. hilgardii* is likely to produce bacterial cellulose, which can considerably increase resistance to the sterilizing agent^10^. In comparison with other wine bacteria such as *Pediococcus pentosaceus* and *Oenococcus oeni*, *L. hilgardii* exhibits higher resistance to flavonoids and phenolic acids^11^. High resistance of *L. hilgardii* to stress conditions is a huge problem in winemaking because elimination thereof from the product is difficult. Although the classic thermal methods of food preservation are still very important in food technology, heat processes can negatively affect the unique properties of product such as taste, colour, and flavour^2^. Scientists around the world are paying special attention to non-thermal methods that will eliminate undesirable microorganisms but will not significantly affect the final stability of the product^3^.

Non-thermal plasma (NTP), also known as cold plasma (CP), is one of the technologies for non-thermal food preservation with great potential as a new decontamination technique. The term cold plasma was first used by Irving Langmuir in 1928 and refers to a partially or fully ionized gas, which consists of molecules in the excited and basic state, reactive compounds, charged particles and UV photons^12^. A number of available publications indicate effective elimination of undesirable microorganisms from food products^13–15^. This wide range of cold plasma applications is related to its ability to inactivate viruses^16,17^, bacteria^18^, spores^19^, yeasts^20^ and fungi^21^. Nevertheless, the exact mechanisms of cold plasma antimicrobial activity are still unknown. Numerous publications indicate that reactive nitrogen and oxygen compounds, high-energy electrons, ionized atoms and particles as well as UV photons present in the plasma stream damage the external structures of microorganisms, have a negative effect on internal cellular components and cause DNA degradation^15,22^. The integrity and proper functioning of the cell membrane of microorganisms is mainly affected by oxidative stress and probably also as some scientists indicate, by generated electrostatic forces. Reactive compounds, mainly oxygen, cause the oxidation of lipids and proteins contained in the membrane, while charged particles accumulating on the outer side of the membrane cause the interruption of its continuity^14,22^. In addition, under the influence of an electric field, an electroporation process may occur, which manifests itself in the formation of micropores^15^. The resulting damage to external cell structures may lead to the leakage of intracellular components. On the other hand, the action of UV photons may cause the formation of nitrogen base dimers, thus disturbing the DNA replication process^14,22^. However, it is known from the available studies that the effectiveness of cold plasma depends on many factors, e.g. operating condition (reactor type, flow, gas pressure, power of plasma excitation, gas type, process duration), exposure mode (direct or indirect), environmental conditions (pH, humidity, matrix type) and the individual morphological and physiological characteristics of the cell^23,24^. Thus, it is necessary to optimize the conditions of plasma treatment in order to make the sterilization with this method effective. Nevertheless, CP has many advantages over conventional techniques, such as the process temperature, which is close to the ambient temperature and therefore does not adversely affect the properties of the product. Furthermore, compared to conventional methods, it is environmentally friendly and cost efficient due to water saving^25^. However, due to the generation of oxidative stress in the cell and the action of an electric field, cold plasma may induce some bacteria to enter the viable but non-culturable (VBNC) stage. Bacteria in this state are characterized by an inability to grow on microbiological media despite their physiological and metabolic activity. The classical plate culture method is most frequently used to determine the microbiological quality of the product. However, this technique does not detect microorganisms that have entered the VBNC state. This is a critical problem in food technology causing adulterated results. Therefore, it is necessary to apply methods that will allow determination of a reliable influence of the new non-thermal decontamination technique on microbial cells^26^.

In this study, inactivation of *L. hilgardii* by cold atmospheric plasma was investigated in a model experimental system. To ensure that the effectiveness of the method was not misinterpreted and damaged cells could be detected, their survival was compared by classical culture, optical density measurement and flow cytometry. Morphological and physiological changes of plasma treated cells were analysed using scanning electron microscopy, atomic force microscopy and Fourier transform-infrared spectroscopy.

## Materials and methods

### Bacterial strains and growth

The *L. hilgardii* strain (NRRL B-1843) was grown overnight (18 h) in MRS (OXOID LTD., Hampshire, England) at 30 °C. Cells were harvested by centrifugation at 8000×*g* for 15 min. The cell pellet was washed once with sterile phosphate-buffered saline (PBS pH 7.4) and twice with sterile water. The pellet was resuspended in sterile water, the bacterial density was measured with a spectrophotometer (SmartSpec Plus Spectrophotometr, USA) at 600 nm, and the number of cells was estimated at 10^8^ CFU mL^−1^ according to the previously established growth curve.

### Experimental design

In order to establish the experimental conditions, preliminary studies were carried out to assess the efficacy of cold plasma inactivation of *L. hilgardii* cells using various critical plasma parameters such as the type of plasma generator (with different configurations and power supplies) and the type of gas used. For the verification tests, two electrode gliding arc (GAD) reactor^27,28^, radio-frequency (RF) plasma jet^29,30^, dielectric barrier discharge (DBD) plasma jet with two ring electrodes on the ceramic tube^31,32^ and DBD plasma jet with one ring electrode on the ceramic tube and grounded electrode below the container were used. After the exposure for 2.5 min, the most effective gas mixture was selected and the operating time was extended to 5 min only for this single gas mixture and the selected reactor. The impact of process parameters was assessed by determining the survival of cold plasma-treated cells were determined by standard counting method on MRS plates. Colonies on plates were enumerated after 6 days incubation at 30 °C, and viable counts were expressed as CFU/mL, as averaged values. Each experiment was performed in triplicate. Finally, process parameters were selected from the obtained data.

### Cold plasma treatment of bacteria

#### Exposure of microorganisms to cold plasma

Four sterile glass tubes containing 6 mL of *L. hilgardii* suspension (10^8^ CFU mL^−1^) in sterile water were prepared. One of them was a control sample not treated with cold plasma. The others were subjected to non-thermal plasma exposure for 5, 10, and 15 min respectively. After that, the samples were transferred to sterile falcons. Part of the research material was immediately used for bacterial growth analysis and SEM imaging. The rest of the material was frozen in dry ice with 20% glycerol and stored at − 80 °C for further experiments.

#### Plasma treatment system

**Figure 1:**
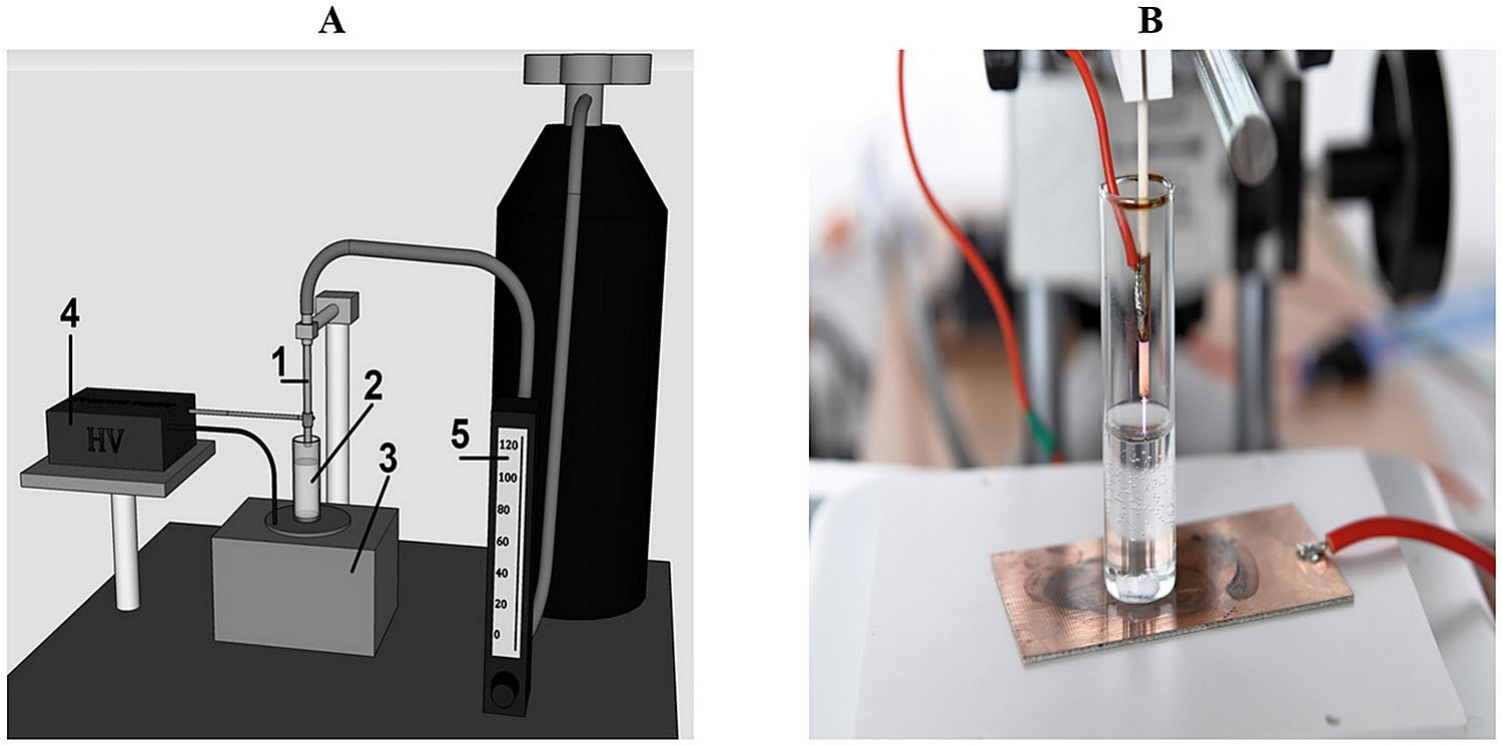
Experimental set-up for plasma treatment: 1—plasma jet reactor; 2—sample in a glass test tube; 3—magnetic stirrer; 4—high voltage power supply; 5—gas flow controller (**A** scheme of experimental set-up; **B** photo of reactor).

**Figure 2:**
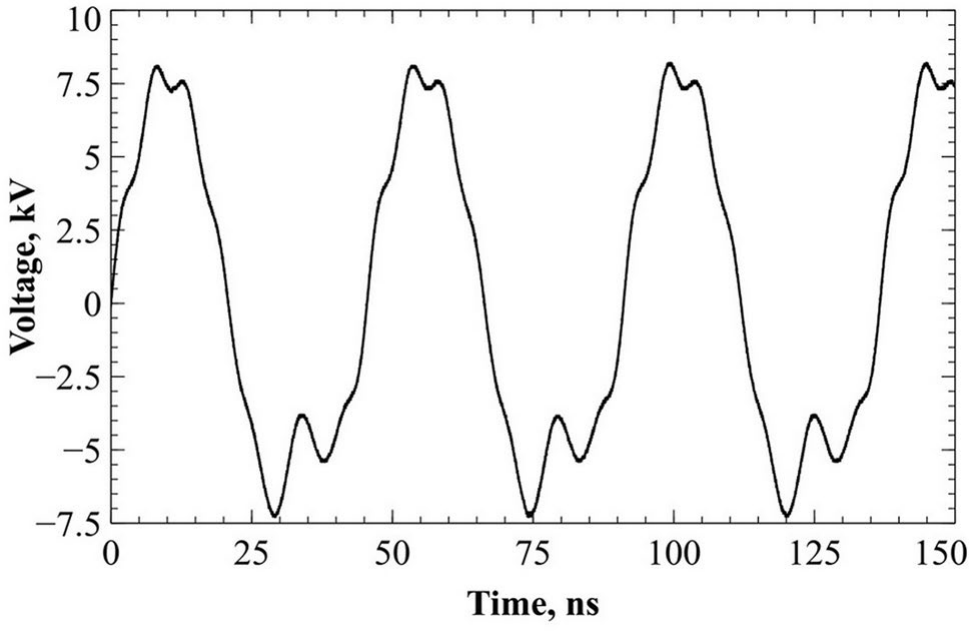
Voltage signal between electrodes.

The cold plasma treatment was performed using a dielectric barrier discharge (DBD) reactor with the power supply of push–pull topology. Helium and oxygen were used as working gases. The atmospheric pressure plasma generated in the reactor had a form of a jet emerging from a ceramic tube with internal and external diameters of 1.4 mm and 3.4 mm, respectively. A high-voltage ring electrode was located 10 mm from the end of the tube. A grounded flat electrode made of copper PCB laminate was placed on a magnetic stirrer. 6 mL of a liquid sample were placed in a glass test tube with a 20-mm internal diameter, directly in the discharge area between the two electrodes. The distance between the end of the reactor and the surface of the liquid was 2 mm. The samples were mixed during the exposure to cold plasma with a PTFE stir bar (Fig. 1). The flow rate of the gas mixture of 96 L/h of helium with 1.8 L/h of oxygen was adjusted by gas flow controllers. The reactor was supplied by RMS voltage of 5.1 kV with frequency of 21.43 kHz and mean power of 6 W. The sine-like voltage signal is shown in Fig. 2.

### Bacterial growth parameters

Growth parameters of *L. hilgardii* before and after the plasma treatment were determined by plate counting method and measurements of optical density (OD_600_) in microcultures with a Bioscreen C system (Labsystem, Helsinki, Finland). Briefly, bacterial cultures were centrifuged and suspended in physiological saline and set to the same optical density of 0.2 at 600 nm. Bacteria were grown in 250 μL of MRS medium, which was transferred into 100-well Bioscreen plates in triplicate, and inoculated with 50 μL of the bacterial suspension. The experiment was performed in aerobic conditions by measuring the OD_600_ every 2 h for 96 h. The following growth curve parameters (i.e. lag time, doubling time, max specific growth rate) were determined using the PYTHON script according to Hoeflinger et al.^33^.

#### Flow cytometry

Cellular metabolic activity and viability of microbial cells from the treated and non-treated samples (10^8^ CFU mL^−1^) were examined using a BacLight Redox Sensor Green Kit (Life Technologies, Carlsbad, CA, USA) and a flow cytometer (cell sorter) BD FACS Aria III (Becton Dickinson, San Jose, CA, USA). Preparation of samples together with configuration and settings of the instrument were based on the protocol described in Duber et al.^34^. The BacLight Redox Sensor Green Kit contains RedoxSensor Green reagent and propidium iodide. The RedoxSensor Green reagent is a fluorogenic redox indicator dye^35^. This compound is subjected to conversion by microbial reductases involved in the electron transport chain. Following excitation (maximum = 490 nm), the converted dye emits green fluorescence (maximum = 520 nm). The intensity of green fluorescence emission is directly proportional to the cellular redox potential (CRP), indicating the levels of metabolic activity of microbial cells^36^. Propidium iodide (PI) is an indicator of cellular integrity facilitating discrimination of live and dead microbial cells. The microbial cells were characterized by two non-fluorescent parameters: forward scatter (FSC) and side scatter (SSC) and two fluorescent parameters: green fluorescence (FITC) from the RedoxSensor Green reagent collected using a 530/30 band pass filter and red fluorescence (PE-TexasRed) from the propidium iodide (PI) reagent collected using a 616/23 band pass filter. For excitation of both fluorescent reagents, 488-nm blue laser was employed. Data were acquired in a four-decade logarithmic scale as area signals (FSC-A, SSC-A, FITC-A, and PE-TexasRed -A) and analysed with FACS DIVA software (Becton Dickinson).

The analysis facilitated detection of dead, mid-active, and active microbial cells. Sub-populations Q1, Q2, and Q4, i.e. dead, mid-active, and active microbial cells, respectively, were selected based on differences in the level of metabolic activity measured as the cellular redox potential (CRP)^37,38^. The definition of the discrete sub-populations was supported by gating in the dot plots of green fluorescence (FITC-A) versus side scatter (PE-TexasRed-A) using quartile gates. The calculation of CRP values was performed using medians of green fluorescence (FITC-A) signals of gated sub-populations.

Microbial cells from the defined sub-populations (Q1, Q2, and Q4) were isolated using a BD FACS Aria III cell sorter. Cell sorting was preceded by the doublet discrimination procedure with the use of height versus width scatter signal measurement (SSC-H vs. SSC-W and FSC-H vs. FSC-W) in order to discriminate single cells from conglomerates allowing high purity sorting. AccuDrop beads (Becton Dickinson, San Jose, CA, USA) were used to setup the drop delay for the best sorting performance. The FACS Aria III cell sorter settings were also established so as to achieve the highest purity level. The cells were sorted to 5 mL cytometry tubes.

#### Recovery of the subpopulation cells

The recovery method of Liu et al. (2018) was used with minor modifications^39^. Cells, which had previously been sorted using flow cytometry into subpopulations in different physiological states (active, mid-active, and dead cells) and stored at − 80 °C with cryoprotectant, were resuspended in fresh sterilized MRS broth supplemented with Tween-20 and amino acids was performed for recovery of potential VBNC *L. hilgardii* cells. Bacteria were grown in 250 μL of MRS medium, which was transferred into 100-well Bioscreen plates in triplicate, and inoculated with 50 μL of the bacterial suspension. The experiment was performed in aerobic conditions by measuring the OD_600_ every 4 h for 168 h. Next one hundred microliters of *L. hilgardii* cells were plated onto supplemented MRS agar. After incubation at 32 °C, microbial growth and colony formation on agar plate was observed.

### Detection of morphological changes

#### Scanning electron microscopy (SEM)

*L. hilgardii* cells (10^8^ CFU mL^−1^) before and after the cold plasma treatment were subjected to SEM analysis in order to investigate morphological changes in the cell surface due to potential cell damage. Bacteria were transferred directly from the culture onto microscopic stubs, frozen in liquid nitrogen, and transferred to the cryo system (Polaron Range, PP 7480). The samples were sublimed (15 min, − 85 °C), sputter-coated with platinum, and transferred into the microscope chamber. The bacteria were observed with the use of an in-lens detector, EHT 3 kV, SEM (Zeiss, ULTRA PLUS).

#### Atomic force microscopy (AFM)

Control samples and samples treated with cold plasma for 10 min, which had previously been sorted using flow cytometry into subpopulations in different physiological states (active, mid-active, and dead cells), were analysed using AFM. After sorting concentration of cells in each samples were respectively; 10^4^ CFU mL^−1^ (active cells), 10^5^ CFU mL^−1^ (mid-active cells) and 10^5^ CFU mL^−1^ (dead cells). Samples were prepared according to Zdybicka-Barabas et al.^40^ and Rachwał et al.^41^. After three rounds of centrifugation (8000*g*, 10 min, 4 °C) of the cells in sterile water, the pellets were resuspended in sterile water at OD_600_ = 0.1. Next, 5 μL of the bacterial suspension were applied to the centre of mica discs and allowed to dry overnight at room temperature. NanoScope Analysis vet. 1.40 software V AFM (Veeco Oyster Bay, NY, USA) was used for imaging the bacterial cell surface (Analytical Laboratory, Faculty of Chemistry, UMCS, Lublin, Poland). The measurements were performed in the ‘Peak Force QNM’ operation mode using a silicon tip with a spring constant of 24 N m^−1^ (NSG30, NT-MDT, Russia) and resonance frequency of 300 kHz. The resolution of the scans was 256 × 256. Three bacterial cells from each mica disc were analysed. The topographies of the tested samples were determined on the basis of height and peak force error images, the DMT module, and adhesion forces between the cell surface and the tip. The data were analysed with Nanoscope Analysis ver. 1.40 software (Veeco, Oyster Bay, NY, USA). The values of average root-mean-square (RMS) roughness and adhesion forces of the cell surface were calculated using 30 fields (120 × 120 nm) in 1 × 1 μm images of the bacterial cell surface. The Student’s *t* test was used to establish the differences between the control samples and bacterial cells in the active, mid-active, and dead states after the 10-min plasma treatment, and P values of < 0.05 were considered significant. The three-dimensional images and section profiles of the *L. hilgardii* cells were generated using Nanoscope Analysis.

#### Fourier transform-infrared spectroscopy (FT-IR)

FT-IR spectra were collected on a Nicolet 8700 FT-IR spectrometer (Thermo Scientific, Waltham, MA, USA) equipped with a KBr beam-splitter and a Mercury Cadmium Telluride MCT/A detector in the transreflection mode. The spectra were recorded over the range of 4000–650 cm^−1^. Each spectrum represented an average of 120 scans and was apodized with the Happ–Genzel function; the number of scan points was 8480. The spectral resolution was 8 cm^−1^. Each sample was measured five times. Each spectrum was baseline corrected and then the spectra were normalized. Assignment of the functional groups of the FT-IR spectra was carried out according to Naumann et al.^42^. To prepare the samples, bacteria (10^8^ CFU mL^−1^) were ground on an aluminium-coated microscope glass slide. The background spectrum was recorded for every sample. FTIR spectra were measured and analysed using Atlus Microscopy Software for OMNIC-8.

## Results and discussion

### Effect of non-thermal plasma on inactivation of *L. hilgardii* cells

The inactivation mechanisms of cold plasma on microbial cells has been confirmed by numerous scientific reports^14,43^. However, there are no studies describing the effects of this sterilizing agent on cells of wine spoilage bacteria such as *L. hilgardii*. Previous reports indicate that the effectiveness of cold plasma inactivation is influenced not only by the environmental conditions (matrix type, pH) and the process parameters (type of reactor, duration of the process) but also by the specific properties of microorganisms^14,15,44^. For this reason, the first step of our experiment was to carry out initial optimization of the process conditions, which included the selection of the reactor, working gas and duration of the plasma treatment of *L. hilgardii* cells. Based on the survival of bacterial cells exposed to cold plasma in various conditions, we decided to use a dielectric barrier discharging cold atmospheric plasma with a mixture of helium and oxygen as working gases in the next stages of the experiment (Table 1).

**Table 1:** Number of live cells of *Lentilactobacillus hilgardii* after cold plasma treatment for 2.5 min and 5 min using various plasma generators (*nt* not tested).

Option	Plasma generator	Gas used	Log N (CFU/mL) 2.5 min	Log N (CFU/mL) 5 min
A	Glide arc	Air	8.86 ± 0.21	Nt
		Nitrogen	8.83 ± 0.15	Nt
		Helium/oxygen	8.83 ± 0.09	7.78 ± 0.07
B	Plasma jet	Air	8.83 ± 0.17	Nt
		Nitrogen	8.81 ± 0.25	Nt
		Helium/oxygen	8.72 ± 0.18	7.28 ± 0.12
C	DBD	Air	8.45 ± 0.09	Nt
		Nitrogen	8.40 ± 0.12	Nt
		Helium/oxygen	7.89 ± 0.05	6.04 ± 0.09
D	RF	air	8.57 ± 0.07	Nt
		nitrogen	8.58 ± 0.13	Nt
		helium/oxygen	7.93 ± 0.21	6.93 ± 0.11

Cell concentrations for control samples at each stage of the experiment were 8.89 ± 0.11 (2.5 min) and 7.81 ± 0.19 (5 min) log CFU/mL respectively.

*DBD* dielectric barrier discharge, *RF* radio frequency discharge.

**Figure 3:**
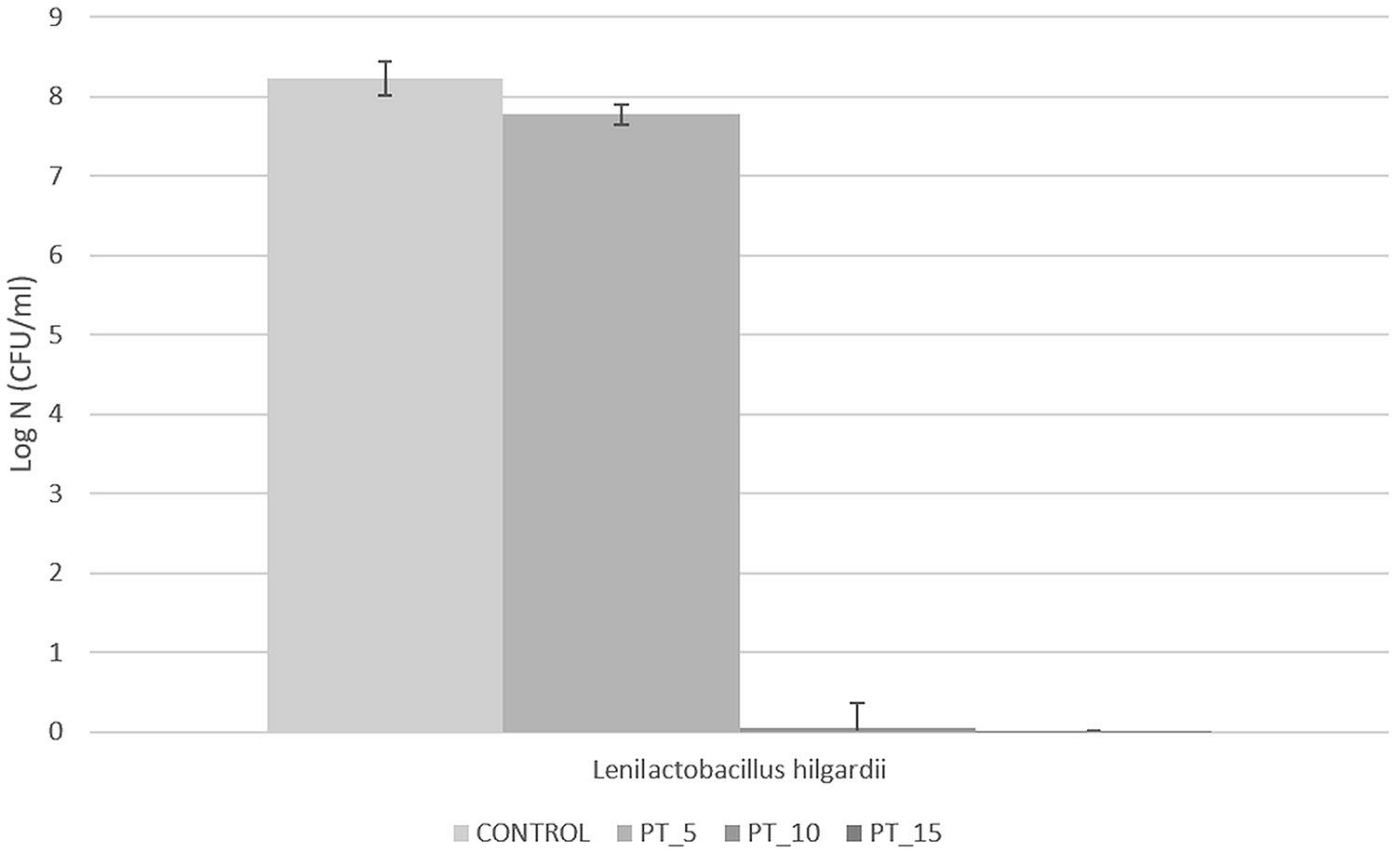
*L. hilgardii* strains population reduction under treatment of DBD. Bars represent standard deviation.

**Figure 4:**
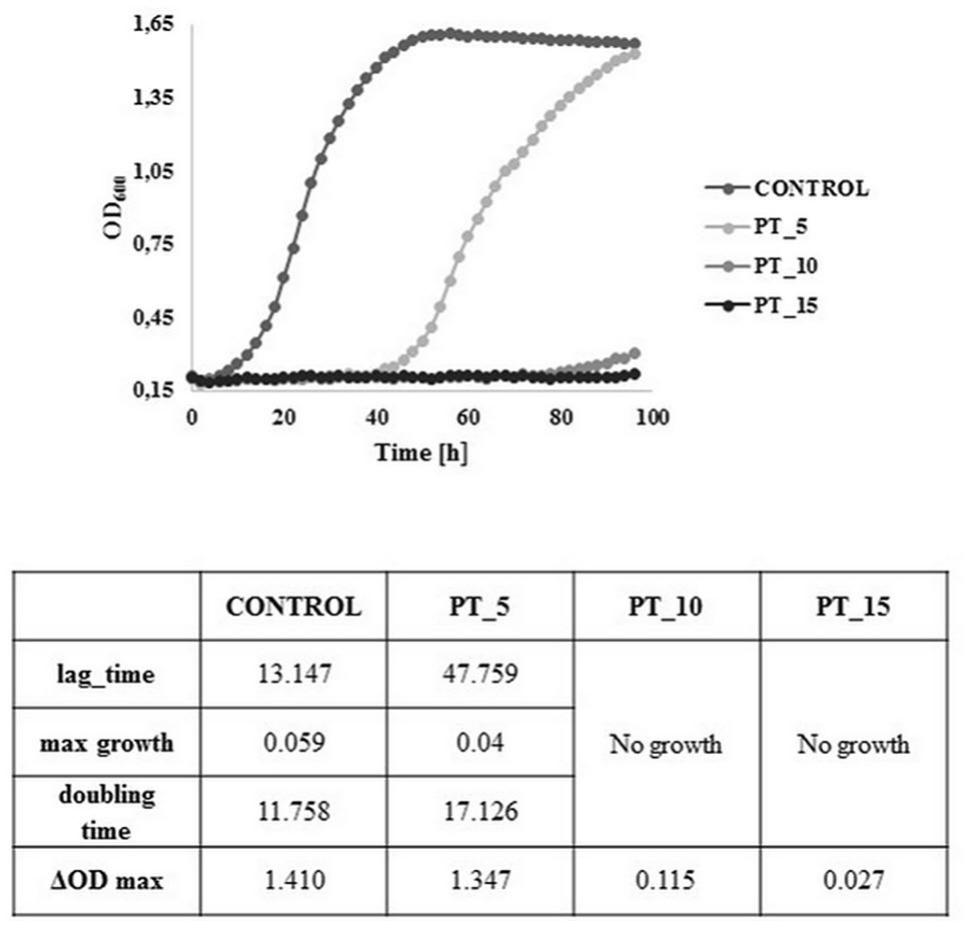
*L. hilgardii* growth parameters after plasma treatment (*PT_5* 5-min plasma treatment, *PT_10* 10-min plasma treatment, *PT_15* 15-min plasma treatment).

**Figure 5:**
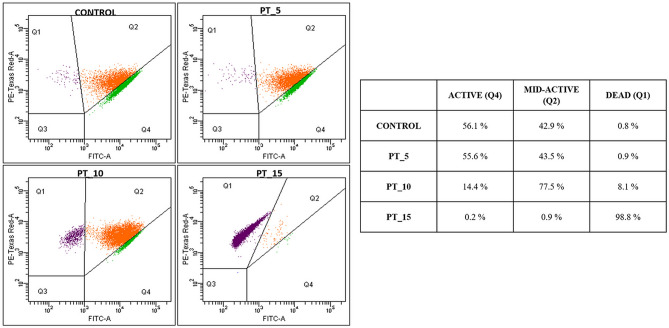
Flow cytometry analysis of the physiological state of *L. hilgardii* cells (active, mid-active, dead) after exposure to cold plasma (*PT_5* 5-min plasma treatment, *PT_10* 10-min plasma treatment, *PT_15* 15-min plasma treatment).

**Figure 6:**
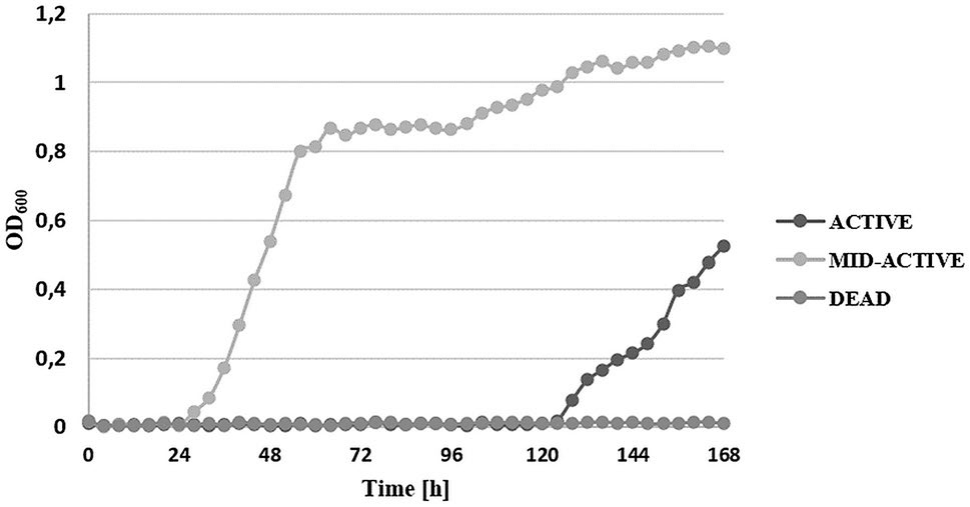
Recovery of the subpopulation *L. hilgardii* cells after 10-min plasma treatment.

An important point in understanding the exact mechanism of the cold plasma action on bacterial cells is to determine the time required for complete growth inhibition^45^. For this purpose, the present study was to evaluated the differences between the application of traditional cells counting techniques and FCM analysis. Experimental data for the *L. hilgardii* growth parameters after cold plasma exposure were determined in comparison with the control sample using the classical plate culture method, optical density measurement and flow cytometry techniques. The inhibition of *L. hilgardii* growth after non-thermal plasma treatment for 5, 10, and 15 min is presented in Figs. 3 and 4. Complete inhibition of bacterial growth was observed after the 10-min cold plasma exposure. However, the 5-min plasma treatment resulted in 263% lag time extension and 46% doubling time extension, compared to the control samples. The distinct lag phase suggested adaption to the stress growth conditions and the onset of repair mechanisms. Since the working gas used was a mixture of helium and oxygen, a high concentration of reactive oxygen species, i.e. singlet oxygen, ozone, hydrogen peroxide, or superoxide anion, was probably present in the plasma stream. For this reason, we suspect that ROS induced oxidative stress in the cell^46^. Nevertheless, lactic acid bacteria have developed a number of defensive mechanisms against ROS. These mainly include excessive production of antioxidant enzymes such as peroxide dismutase (peroxide degradation), which is encoded by the sodA gene and its expression occurs in the presence of oxygen^47^. However, other studies, in which DBD was also the plasma source and the working gas was an argon/oxygen mixture, showed that the time needed to fully inactivate Gram-negative bacteria (*Pseudomonas aeruginosa, Escherichia coli, Vibrio parahaemolyticus*) and Gram-positive bacteria (*Listeria monocytogenes, Staphylococcus aureus*) was 5 min. Nevertheless, these differences may result from the fact that the number of living cells was determined only by plate counting method^48^. Flow cytometry in combination with specific fluorescent staining facilitated characterization of the physiological states of bacterial cells in the tested samples. The BacLight Redox Sensor Green Vitality Kit (Thermo Scientific) was used to characterize and distinguish the different physiological states of the treated microbial strains based on measurements of cellular metabolic activity. The flow cytometry analysis facilitated discrimination of the discrete sub-populations of active (Q4), mid-active (Q2), and dead (Q1) microbial cells. The interpretation of the results of the experiment consisted in determining the percentages of the defined sub-populations of dead, mid-active, and active cells (assessment of their distribution in the entire population of microorganisms in the tested samples. The percentages of the defined microbial sub-populations demonstrated considerable differences in the distribution of individual sub-populations of active, mid-active, and dead microbial cells (sub-population Q4, Q2, and Q1, respectively) between the untreated (control) and cold plasma-treated samples (Fig. 5). The microbial cells from mid-active sub-population (Q2) may represent the dormant and injured cells (including VBNC), which may undergo a resuscitation process^36^ and regain viability^49^ which leads to the recovery of culturability. The substantial resolution of the applied assay helped to characterize and distinguish the different physiological states to monitor the germicidal effect of the tested anti-microbial procedure. The first indication of the bactericidal effect was demonstrated in the sample subjected to the 10-min plasma exposure (PT_10) as an 8.1% increase in the percentage of dead bacterial cells. This was accompanied by a considerable increase in the percentage of mid-active cells (77.5% vs. 42.9% for the untreated control). The *L. hilgardii* sample subjected to the 15-min cold plasma treatment (PT_15) demonstrated a virtually detrimental effect with the dominance of dead bacterial cells (98.8%). The flow cytometry evaluation of the metabolic activity of the microbial cells helped to discriminate between different physiological states of microbial cells in the plasma-treated samples. This facilitated monitoring of the microbial heterogeneity to evaluate the impact of the tested anti-microbial procedure on the viability and vitality of the microbial community at the single-cell level. As shown by the bacterial growth curve in Fig. 4, *L. hilgardii* subjected to the 10-min plasma treatment exhibited cell growth on the fourth day of culture. Our results demonstrate that cold plasma treatment may have constituted an environmental stress that initiated response strategies in the *L. hilgardii* cells. Pavlov and Ehrenberg have described a model in which bacteria manipulate their own gene expression to adapt quickly to changes in the environment^50^. Microbial response provides bacteria with an ability to concentrate on growth metabolism instead of multiplying under environmental stress^51^. In our experiment, we can assume that the *L. hilgardii* response includes the viable but non-culturable (VBNC) state, in which the bacteria cannot form a colony on solid media but still possess the capability of renewed metabolic activity, namely, to be still alive in favourable conditions. These strategies inevitably pose a potential hazard to the food processing industry. The VBNC state is a bacterial strategy to survive adverse conditions causing cellular stress, i.e. extreme temperatures, elevated osmotic pressure, exposure to light or oxygen content. As mentioned earlier in the plasma stream, whose working gas is oxygen, the ROS concentration is high, which can potentially induce cell entry into the VBNC state^46^. In order to confirm our hypothesis that cold plasma induces VBNC state in *L. hilgardii* cells, we have performed resuscitation of subpopulation cells on supplemented MRS broth and MRS agar. Figure 6 shows the growth of the recovery cells. There was observed the growth of active and mid-active cells and the lack of growth of dead cells. This may confirm our assumption that some of the mid-active cells of *L. hilgardii* are able to be cultured under appropriate conditions. The resuscitation experiment was carried out after storing the samples at − 80 °C (with cryoprotectant), therefore we observe a longer stationary phase. It is worth noting the faster growth of cells in the VBNC state in relation to active cells. At this stage of the experiment we can assume that the faster and more intensive cell divisions may be influenced by defense mechanisms activated under environmental stress, such as cold plasma. Although our study did not include investigation of changes in gene expression under the influence of cold plasma, however, on the basis of studies carried out by other authors we can assume that the potential entry into VBNC was related to the loss of catalase activity^52^. Recent studies indicate that *Schleiferilactobacillus harbinensis* (previously *Lactobacillus herbinesis*)^7^ strains from rotten beer can enter the VBNC state and, what is important, the cells recover their ability to grow on media with catalase^46^. Furthermore, other reports confirmed that new non-thermal decontamination techniques such as high hydrostatic pressure (HHP) induced cell entry into the VBNC state^26,53^. Unfortunately, in most literature reports on the efficacy of cold plasma, only a few authors use detection methods that are more sensitive than the plate culture methods^26^. A possibility of induction the VBNC state by cold plasma was reported by Dolezalova and Lukas^54^ who received a 7 log reduction in *E. coli* growth assessed by the plate count as a result of treatment with an atmospheric pressure plasma jet. In turn, the LIVE/DEAD technique showed only 1 log reduction of *E. coli.* These results show that the classical culture methods are insufficient to determine the effectiveness of new sterilization methods and may cause falsification of the results. In addition, cold plasma may induce the VBNC state in *L. hilgardii* cells.

### Effect of cold plasma on bacterial cell morphology

**Figure 7:**
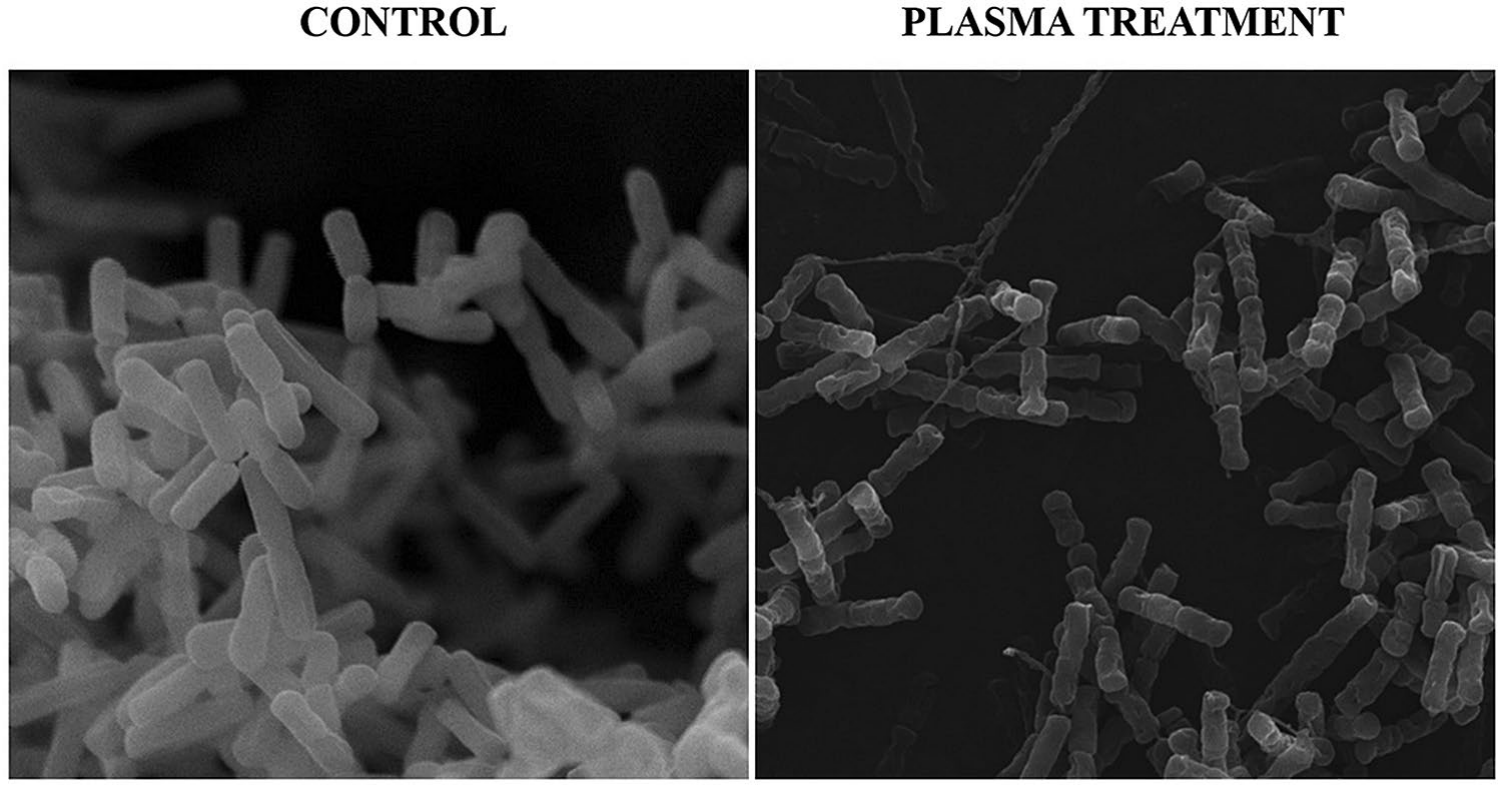
Scanning electron micrographs of *L. hilgardii cells*: untreated (control) and after 10-min cold plasma treatment.

Cold plasma inactivation is primarily based on the interaction of its components with the cell surface. The oxygen and nitrogen compounds in the plasma cause oxidation of lipids and proteins of the membrane and, consequently, interruption of its continuity. Additionally, the plasma contains charged particles, which accumulate on the outer side of the membrane leading to its disintegration^15^. In order to determine the relationship between the physiological state and morphology of the cell after exposure to cold plasma, we used various microscopic techniques to characterize cell wall damage at the nanoscale. Scanning electron microscopy (SEM) was used to examine the cell surface. Atomic force microscopy (AFM) was used to correlate the nanometric scale observations with the nanomechanical and physical properties. The SEM analysis was carried out to observe the morphological changes in plasma-untreated bacterial cells in comparison to the cells treated with the plasma for 10 min (Fig. 7).

**Figure 8:**
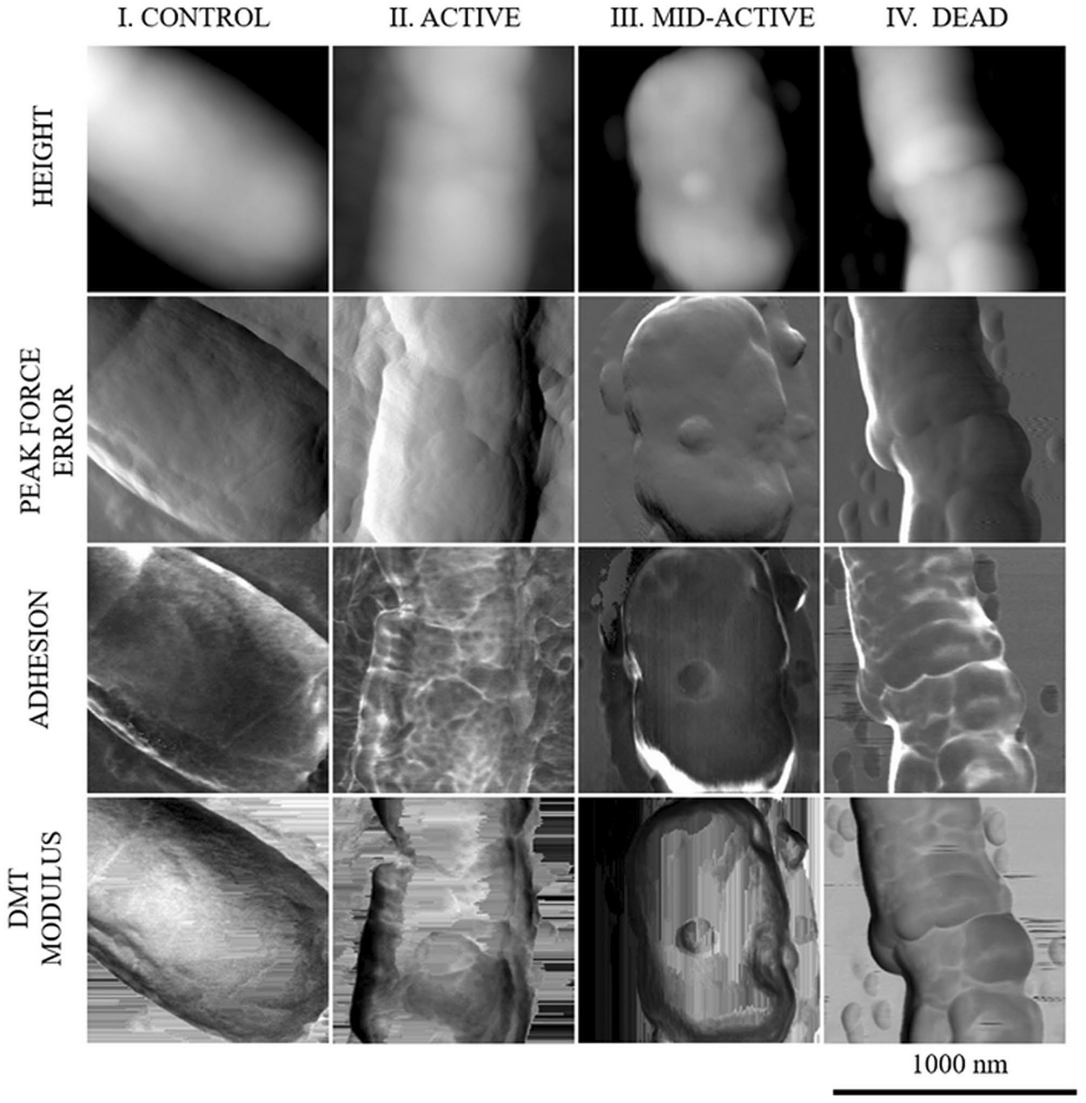
AFM imaging of cells sorted into subpopulations after 10-min plasma treatment.

**Figure 9:**
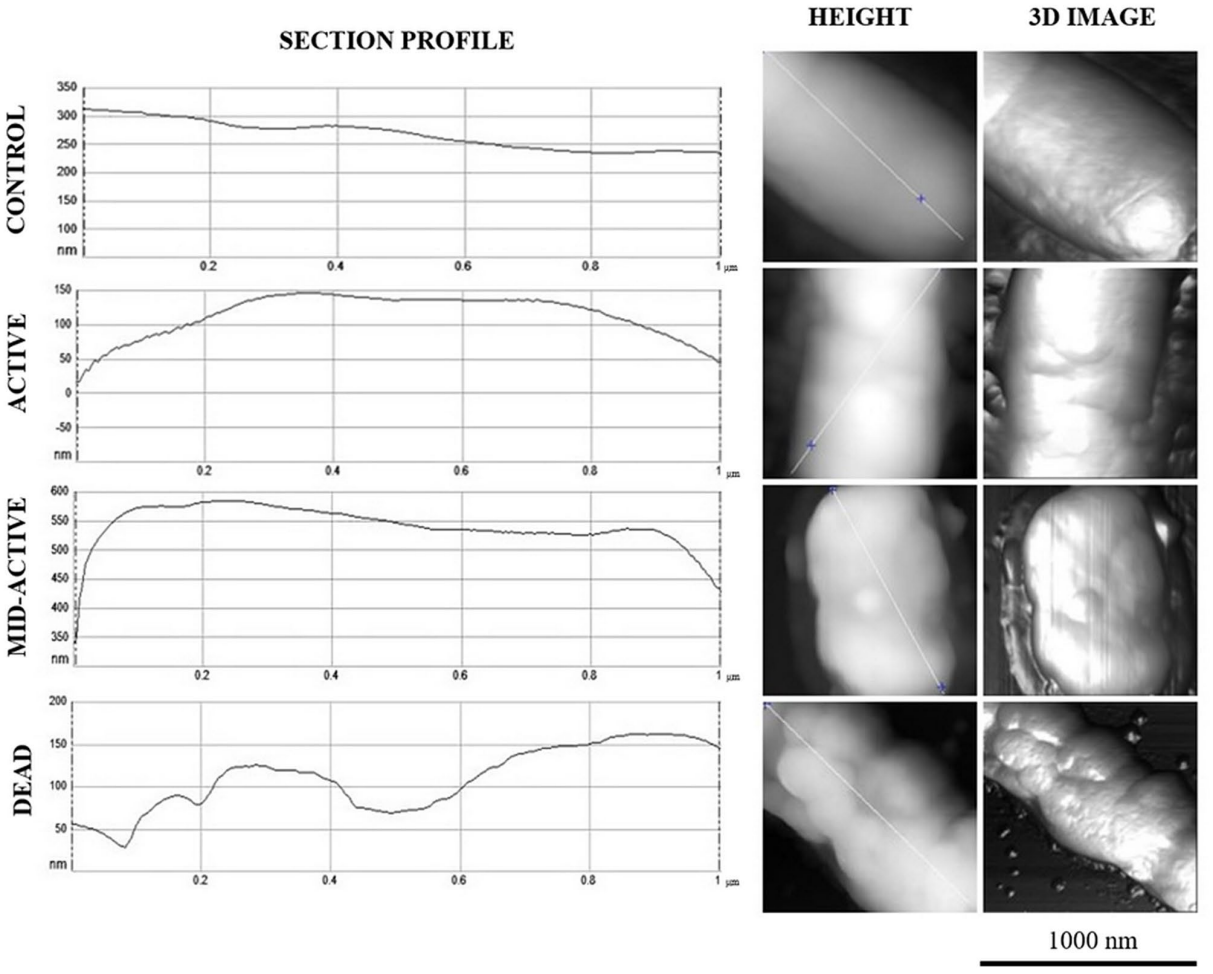
Section profile of the cell surface of *L. hilgardii* cells in three different physiological states after plasma treatment.

The untreated *L. hilgardii* cells are visible as short or long rod-shaped cells with a smooth surface. In contrast, most of the treated cells have an irregular shape with deep cavities on the surface. In some cells, the outer layer looks as if it has been etched. This effect is probably caused by radicals (OH^−^ or NO^−^) that cause erosion and digestion of the cell membrane^26^. However, the substantial number of cells in the intermediate state indicates that not all cells have suffered sufficiently serious external damage that would lead to their death. Previous studies have pointed out differences in the action of cold plasma on bacterial cells due to the different structure of external cellular covers. In general, Gram-positive bacteria are considered to be more resistant to CP than Gram-negative species, and the mechanism of their inactivation based on reactions of ROS (reactive oxygen species) will cause severe damage to intracellular components (e.g., DNA) but not cell leakage^55^. In our study, atomic force microscopy (AFM) images were used for further investigations of the changes in *L. hilgardii* before and after the CP treatment. The analysis allowed determination of the discrepancies in the topography and surface properties of cells after the 10-min exposure to cold plasma in three different physiological states (active, mid-active, dead) compared with cells that were not exposed to the sterilizing agent. To the best of our knowledge, AFM was used for the first time in this work to evaluate cells sorted into subpopulations after cold plasma treatment. Cells in the control sample had a typical rod shape and smooth surface, whereas cells subjected to the 10-min plasma treatment (active, mid-active) had a more irregular shape, which can be seen as the height and peak force error images (Fig. 8). The cells in the intermediate state were shorter and smaller. Many authors point out a marked reduction in the cell size in the VBNC state (shorter length), which is a starvation survival strategy^56,57^. Interestingly, the section profile (Fig. 9) also indicates that these cells were almost twice the height of the control cells. Similar results were obtained by Hung et al.^58^, who observed *Vibrio parahaemolyticus* in the VBNC state as bulged cells. The transition from rod-shaped bacterial morphology into coccoid morphology has been characterized for some bacteria in the literature^59,60^. A change in the shape is likely to contribute positively to the uptake of nutrients by the cell. This is related to the large cell surface area compared to the cellular volume^61^. However, considerable differences in cell topography can be observed in cells whose physiological condition indicates that they are dead. The section profile (Fig. 9) indicated that these cells had deep, irregular depressions on their surface but it seemed that the integrity of the outer layers was not interrupted. This is consistent with previous reports demonstrating that inactivation of Gram-positive bacteria is the result of ROS action on the internal components of cells. Moreover, the nanomechanical properties of cells in the examined physiological states in comparison with the control are shown in the peak force error, DMT module, and adhesion images. Thanks to the application of AFM technique, in addition to images showing the differences in the cell surface, we obtained figures that allowed us to calculate values corresponding to cell surface RMS roughness, adhesion, elasticity, and stiffness (Table 2).

**Table 2:** Values of DMT Modules, roughness and adhesion of cells surface sorted into subpopulations after 10-min plasma treatment.

	Control	Active	Mid-active	Dead
DMT modules	1.81 ± 0.24 GPa	1.97 ± 0.19 GPa	2.19 ± 0.17 GPa	1.45 ± 0.28 GPa
Roughness	2.04 ± 0.55 nm	2.11 ± 0.57 nm	2.70 ± 0.75 nm	7.07 ± 3.1 nm
Adhesion	0.32 ± 0.08 nN	0.29 ± 0.09 nN	0.43 ± 0.09 nN	0.24 ± 0.02 nN

The DMT Module in the dead cells was 1.45 ± 0.28 GPa, which indicated that the surface was more flexible than surface of control cells (1.81 ± 0.24 GPa) after the plasma treatment. In turn, the highest stiffness (2.19 ± 0.17 GPa) was typical for cells in the intermediate state; however, the results were similar to those of the control and living cells. An increase in stiffness is probably associated with a change in the cell shape. Research conducted by Vattakaven et al.^62^ showed that *Vibrio tasmaniensis* in the VBNC state changed into coccal forms with a thicker cell wall after 150-day cold- starvation^62^. In addition, the roughness of the dead cells (7.07 ± 3.1 nm) was statistical significantly higher in relation to the control (2.04 ± 0.55 nm) (p < 0.05). The enhanced roughness suggests disorganization of the cell wall surface and correlates with the changes observed by SEM. The roughness of the living cells should be underlined. Despite the exposure to cold plasma, the roughness of their outer structures was almost the same as that of the cells in the control sample. These unchanged cell surface properties in the viable and intermediate states suggest that some of mid-active bacteria can resume growth and divisions in favourable environmental conditions.

#### FT-IR

**Figure 10:**
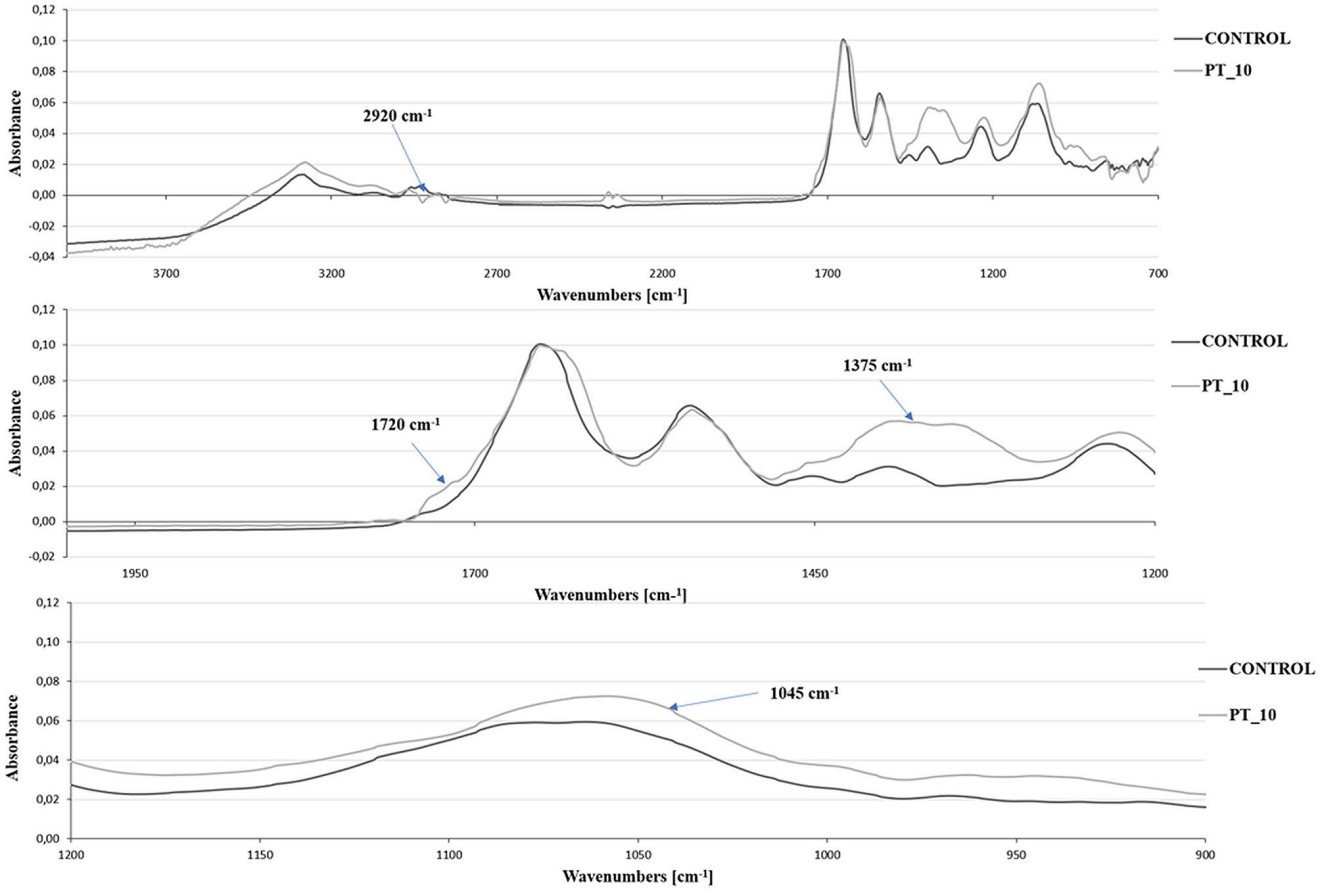
FT-IR spectra of *L. hilgardii* cells: untreated (control) and after 10-min cold plasma treatment.

The analysis of the chemical modifications in the outer structure of plasma-treated *L. hilgardii* bacteria was performed using the FT-IR technique. A comparison of the control sample with the sample subjected to 10-min cold plasma exposure demonstrates a substantial number of spectral changes associated with vibrations of functional groups such as carbohydrates, fatty acids, proteins, or nucleic acids (Fig. 10). The 3100–2800 cm^−1^ spectral region is the fatty acid region, 1800–1500 cm^−1^ represents the amide region, 1500–1200 cm^−1^ is a mixed region containing signals from proteins, fatty acids, and phosphate-carrying compounds, 1200–900 cm^−1^ is the polysaccharide region, and 900–700 cm^−1^ is the “fingerprint region”^63^. In first region, a decrease around the bands ~ 2960 cm^−1^ and 2920 cm^−1^ was observed after the plasma treatment, which is related to destruction of antisymmetric stretching in the CH_3_ and CH_2_ groups of fatty acids. Similar results were presented by Khan et al.^64^, who examined the effect of DBD on the mechanism of bacterial biofilm inactivation. The authors suggested that the decrease in the intensity of the spectra corresponded to cell membrane disturbances^64^. The FT-IR investigations show very prominent increase around 1375 cm^−1^ which may be related to symmetric stretching vibration of C=O indicating a formation of the carboxylate groups (COO^-^). Figure 10 shows a rising shoulder at 1720 cm^−1^, indicating the formation of new carbonyl groups like aldehydes, ketones, or acids. Probably, the occurrence of these two signals is associated with the degradation of the cell wall and membrane caused by ROS (e.g. OH^−^ and O^2−^)^65,66^. There is also an increase in the band at 1045 cm^−1^. As described in other reports, protein treatment with CAP is probably associated with symmetrical tensile vibrations of S=O and radical oxidation of sulphur-containing amino acid methionine^67–69^. This demonstrates that plasma can cause oxidation of sulphur-containing molecules such as amino acids (methionine, cysteine), proteins, or compounds with antioxidant effects, e.g. glutathione (GSH).

## Conclusion

In this work a model experimental system was used to assess the effect of cold plasma on *L. hilgardii* cells. Differences between cell viability results obtained by plate count method, optical density measurement and flow cytometry are presented. In the classical plate culture method and optical density measurement complete inhibition of bacterial growth was observed after the 10 min cold plasma exposure. On the other hand, flow cytometry analysis indicating the levels of metabolic activity of microbial cells revealed the presence of three physiological states: active (14.4%), mid-active (77.5%) and dead (98.8%) of *L. hilgardii* population after 10 min plasma treatment. Re-cultivation of the sorted subpopulation seems to confirm that at least some of the mid active cells were in the VBNC state. Moreover, application of AFM technique indicated that morphological changes in external cell structures in the intermediate state did not show any considerable damage compared to control cells. This also suggests that cells can grow again in favorable conditions. However, entry of the food spoilage bacteria in VBNC state is a potential threat to the food industry. The classical plate culture method, which is not able to detect the mid-active bacteria, is commonly used to determine the microbiological purity of a food product. For new non-thermal food preservation methods such us cold plasma, whose exact mechanism of microbial inactivation is not yet known, it is necessary to use more sensitive detection methods that will give a reliable result on the microbiological purity of the product. In summary, the flow cytometry used in this work, combined with cell sorting that allows the analysis of different subpopulations, is a valuable new approach in explaining the bactericidal effect of cold plasma and requires further research will help explain the exact mechanism of microbiological inactivation in the future.
